# Individual Neurons Confined to Distinct Antennal-Lobe Tracts in the Heliothine Moth: Morphological Characteristics and Global Projection Patterns

**DOI:** 10.3389/fnana.2016.00101

**Published:** 2016-10-24

**Authors:** Elena Ian, Xin C. Zhao, Andreas Lande, Bente G. Berg

**Affiliations:** ^1^Department of Psychology, Norwegian University of Science and TechnologyTrondheim, Norway; ^2^Department of Entomology, College of Plant Protection, Henan Agricultural UniversityZhengzhou, China

**Keywords:** central olfactory pathway, antennal-lobe projection neurons, iontophoretic intracellular staining, confocal imaging, 3D reconstruction, standard brain atlas

## Abstract

To explore fundamental principles characterizing chemosensory information processing, we have identified antennal-lobe projection neurons in the heliothine moth, including several neuron types not previously described. Generally, odor information is conveyed from the primary olfactory center of the moth brain, the antennal lobe, to higher brain centers via projection neuron axons passing along several parallel pathways, of which the medial, mediolateral, and lateral antennal-lobe tract are considered the classical ones. Recent data have revealed the projections of the individual tracts more in detail demonstrating three main target regions in the protocerebrum; the calyces are innervated mainly by the medial tract, the superior intermediate protocerebrum by the lateral tract exclusively, and the lateral horn by all tracts. In the present study, we have identified, via iontophoretic intracellular staining combined with confocal microscopy, individual projection neurons confined to the tracts mentioned above, plus two additional ones. Further, using the visualization software AMIRA, we reconstructed the stained neurons and registered the models into a standard brain atlas, which allowed us to compare the termination areas of individual projection neurons both across and within distinct tracts. The data demonstrate a morphological diversity of the projection neurons within distinct tracts. Comparison of the output areas of the neurons confined to the three main tracts in the lateral horn showed overlapping terminal regions for the medial and mediolateral tracts; the lateral tract neurons, on the contrary, targeted mostly other output areas in the protocerebrum.

## Introduction

The present investigation, being a descriptive neuroanatomical study on the olfactory system, is a continuation of pioneering work performed more than a century ago. As early as 1890, Ramón y Cajal described not only the cellular layers in the rat olfactory bulb but also the glomeruli and the neuronal elements forming these synaptic structures (Oñate et al., 2014). Indeed, the exploration of neural architecture and its basic units is essential for any further attempt to understand how a neural system is functioning. The focus of our study is the second level of the moth olfactory pathway – formed by principal antennal-lobe neurons projecting to higher brain centers via several parallel tracts. Although the issue of parallel olfactory processing in both insects and mammals has received considerable attention (Galizia and Rössler, 2010; Igarashi et al., 2012; Rössler and Brill, 2013; Strutz et al., 2014; Carcaud et al., 2016; Mori, 2016) the functional role of such an arrangement is still poorly understood.

Due to the moth’s exquisite sense of smell, combined with its relatively simple and accessible nervous system, this group of nocturnal insects has been appreciated in a body of research essential for understanding chemosensory processing principles. The moth olfactory system has therefore been described in several previous works (reviewed by Anton and Homberg, 1999; Haupt et al., 2010; Martin et al., 2011). As in other insects, olfactory sensory neurons covering the antenna of the moth project directly to the primary olfactory center of the brain, the antennal lobe (AL) - which is the analog to the mammalian olfactory bulb. Here, the sensory axons make synapses with two main types of AL neurons: projection neurons (PNs) carrying signal information to higher integration centers in the protocerebrum and local interneurons confined to the AL. A third and minor category of antennal-lobe elements is the centrifugal neurons conveying feedback information from various regions of the central nervous system to the AL.

Three main tracts in the moth brain, named the medial, the mediolateral, and the lateral antennal-lobe tract (mALT, mlALT, and lALT, respectively), connect the AL with higher integration centers in the protocerebrum, including two prominent regions named the calyces (Ca) of the mushroom bodies and the lateral horn (LH; see ; Homberg et al., 1988; Seki et al., 2005; Rø et al., 2007; Ito et al., 2014). These ALTs, which are formed primarily by axons of AL PNs, correspond to the mammalian olfactory tract targeting regions in the temporal lobe (reviewed by Lledo et al., 2005). Recently, we performed distinct mapping of the ALTs in the heliothine moth, demonstrating the projection pattern of the individual tracts in greater detail (Ian et al., 2016). Among the new findings was that the lALT has an inconspicuous connection with the Ca – which is somewhat different from what was previously reported (Rø et al., 2007; Galizia and Rössler, 2010; Martin et al., 2011). This recent study also emphasized one particular sub-branch of the lALT, a fiber bundle projecting to a pillar-shaped region located between the anterior optic tubercle (AOTU) and the alpha-lobe of the mushroom body. In spite of being reported both by Homberg et al. (1988) and by Rø et al. (2007), the prominence of this sub-branch has sometimes been overlooked. One of a few additional ALTs that have been found both in the sphinx moth, *Manduca sexta*, and in the heliothine moth, *Heliothis virescens*, is the dorso-medial ALT (dmALT) (Homberg et al., 1988; Rø et al., 2007). This tract is reported to include bilateral neurons innervating the calyces and the inferior protocerebrum of both hemispheres (Rø et al., 2007). Besides the tracts mentioned above, a new ALT was discovered in the recent study of the heliothine moth. This transverse ALT (tALT) splits from the mALT at the lateral edge of the central body (CB) and projects laterally to the lateral protocerebrum, however, in a more posterior position compared to the course of the mlALT (Ian et al., 2016). Thus, according to the morphological studies mentioned above, the ALTs in the moth target three main ipsilateral protocerebral regions: (1) the Ca are innervated mainly by the mALT, (2) the region between the AOTU and the alpha-lobe is innervated selectively by a sub-branch of the lALT, and the LH is innervated by several ALTs (Homberg et al., 1988; Seki et al., 2005; Rø et al., 2007; Ian et al., 2016).

A substantial amount of individual ALT PNs that have been morphologically and physiologically characterized in various moth species belong to the mALT PNs (*Agrotis segetum*: Hansson et al., 1994; *Bombyx mori*: Kanzaki et al., 2003; *H. virescens*: Christensen et al., 1995; Berg et al., 1998; *Helicoverpa zea*: Christensen et al., 1991; Vickers et al., 1998; *H. assulta*: Zhao and Berg, 2010; *M. sexta*: Christensen and Hildebrand, 1987; Hansson et al., 1991; *Spodoptera littoralis*: Anton and Hansson, 1994). These uni-glomerular PNs innervate the ipsilateral Ca before terminating in the LH. Medial-tract neurons connected to the male-specific macroglomerular complex (MGC), processing pheromone information, are reported to target a region both in the Ca and in the LH that is distinct from the region innervated by plant odorant neurons (Homberg et al., 1988; Seki et al., 2005; Zhao et al., 2014). In heliothine moths, most male-specific PNs confined to the mALT are reported to display relatively narrowly tuned response profiles (reviewed by Berg et al., 2014).

Pure morphological characterization of individual PNs confined to different ALTs has been performed as well (*M. sexta*: Homberg et al., 1988; *B. mori*: Seki et al., 2005; *H. virescens*: Rø et al., 2007). Generally, these data demonstrate that the mALT consists mainly of a homogenous population of PNs, including the uniglomerular type mentioned above. The thinnest of the classical tracts, the mlALT, projecting directly to the LH without innervating the Ca, is reported to contain multiglomerular PNs. A substantial proportion of these neurons are GABAergic (Hoskins et al., 1986; Iwano and Kanzaki, 2005; Berg et al., 2009; Liang et al., 2013). The PNs confined to the lALT comprise both uni- and multiglomerular PNs. These neurons form a shared fiber bundle at the exit of the AL, but soon proceed along different trajectories. The morphologically different lateral-tract PNs include a few neurons targeting the contralateral protocerebrum as well (Homberg et al., 1988). In other words, the lALT comprises PNs of diverse morphologies and trajectories.

Based on the new findings on the ALTs of the moth, as recently described by Ian et al. (2016), it is relevant to upgrade the overall picture of the olfactory pathways from the AL to the higher levels of the system by adding data on individual PNs confined to the distinct tracts. Moreover, advancement in image acquisition and digital tracing allows visualization of neurons in three-dimensional spaces, enabling comparison of individual neurons as well as characterization of the neurons’ global properties including their putative connectivity maps (Parekh and Ascoli, 2013). Through this paper, we continue and expand the previous work carried out by Homberg et al. (1988) and Rø et al. (2007) by presenting anatomical data on individual AL PNs in the moth brain. Our data include 46 PNs confined to all five tracts mentioned above. In addition to several new morphological types of PNs, we present completely stained neurons, which were formerly visualized only partly or together with other stained neurons. Last but not least, we have reconstructed a selection of the individual PNs and registered the digital models into the standard brain atlas created for the *H. virescens* male (Zhao et al., 2014). By comparing the PNs’ innervation patterns we found that neurons confined to two of the three classical tracts, the medial and the mediolateral tract, target the same region in the lateral horn indicating that they are included in the same neural network. Neurons confined to the lateral tract, on the other hand, split into several paths all targeting different protocerebral regions.

## Materials and Methods

### Insects

Males of *H. virescens* (Lepidoptera: Noctuidae; Heliothinae) were used for the experimental work (three females were included). Eggs were supplied by Bayer Crop Science AG (Mohnheim am Rhein, Germany) and larvae were reared on an artificial diet (Wu and Gong, 1997). Pupae were kept at 25°C and 67% humidity on a phase-shifted LD 14–10 h. Adults, 3–7 days, were used for the experiments. The preparation of the insect has been described previously (Rø et al., 2007; Zhao et al., 2014). Briefly, the moth was restrained inside a plastic tube with the head exposed and then immobilized with dental wax (Kerr Corporation, Romulus, MI, USA). The brain was exposed by opening the head capsule and removing the muscle tissue. The sheath of the antennal lobe was removed by fine forceps in order to facilitate microelectrode insertion into the tissue. The exposed brain was continuously supplied with saline solution (in mM: 150 NaCl, 3 CaCl2, 3 KCl, 25 Sucrose, and 10 N-tris (hydroxymethyl)-methyl-2-amino-ethanesulfonic acid, pH 6.9). According to Norwegian law of animal welfare there are no restrictions regarding experimental use of Lepidoptera.

### Intracellular Staining

The intracellular staining of antennal-lobe PNs was performed as previously described (Zhao et al., 2014). Sharp glass electrodes were pulled with a horizontal puller (P97; Sutter Instruments, Novarto, CA, USA). The tip of the micro-pipette was filled with a fluorescent dye (4% tetramethylrhodamine dextran with biotin, Micro-Ruby, Molecular Probes; Invitrogen, Eugene, OR; in 0.2 M K-acetate) and the glass capillary back-filled with 0.2 M K^+^-acetate. The recording electrode, having a resistance of 150–300 MΩ, was lowered carefully into the AL by means of a micromanipulator (Leica). A chloridized silver wire inserted into the eye served as the reference electrode. When obtaining stable contact with a neuron, 1–2 nA depolarizing current pulses with 200 ms duration at 1 Hz were applied for 10–20 min. To allow neural transportation of the dye to all axonal processes, the preparation was kept overnight at 4°C. The brain was then dissected and fixed in 4% paraformaldehyde for 2 h at room temperature and rinsed with a phosphate-buffered saline (PBS; in mM: 137 NaCl, 2.7 KCl, 10 Na2HPO4, 1.8 KH2PO4; pH 7.4). Some of the preparations were incubated in fluorescent conjugate streptavidin-Cy3 (Jackson Immunoresearch, West Grove; PA, USA, diluted 1:200 in PBS), for 2 h. Finally, the preparation was dehydrated in an ascending ethanol series (50, 70, 90, and 96%, 2x 100%; 10 min each) before being cleared and mounted in methyl salicylate (Sigma–Aldrich, Germany), by means of 0.5 mm thick aluminum slides.

### Immunocytochemistry

In order to visualize neuropil structures in the brain preparations with successfully labeled neurons, an antiserum marking synaptic regions (SYNORF 1) was used in immunostaining experiments. The anti SYNORF 1 (Developmental Studies Hybridoma Bank Univerity of Iowa) was raised against fusion proteins composed of glutathione-*S*-transferase and the *Drosophila* SYN1 protein (SYNORF 1; Klagges et al., 1996), and it is reported to detect synaptic neuropil in various insect species, heliothine moths included (Berg et al., 2002; Kvello et al., 2009). After analyzing the iontophoretically stained neuron by confocal microscopy, the brain was rehydrated through a decreased ethanol series (10 min each) and rinsed in PBS. To minimize non-specific staining, the brain was submerged in 5% normal goat serum (NGS; Sigma, St. Louis, MO, USA) in PBS containing 0.5% Triton X-100 (PBSX; 0.1 M, pH 7.4) for 3 h at room temperature before being incubated in the primary antibody SYNORF1 at 1:100 in PBSX at 4°C for 5 days. Then, the brains were rinsed in PBS for 6 × 20 min before being incubated in the secondary antibody, Cy2-conjugated anti-mouse (dilution 1:300 in PBSX; Invitrogen, Eugene, OR, USA), at 4°C for 3 days. The brains were finally rinsed for 6 × 20 min in PBS, dehydrated with an ascending ethanol series, and mounted in methyl salicylate.

### Confocal Microscopy and Image Processing

All successfully stained neurons were imaged by using a confocal laser scanning microscope (LSM 510 META Zeiss, Jena, Germany) equipped with a Plan-Neofluar 20x/0.5 and C-Achroplan 40x/0.8W objective. The Micro-Ruby (Ex_max_ 555 nm, Em_max_ 580 nm) staining was excited by the 543-nm line of a HeNe 1 laser (filter BP 565-615 IR) and the Cy2 (Ex_max_ 492 nm, Em_max_ 510 nm) by the 488-nm line of an argon laser. The optical sections were scanned with a high resolution (1,024 pixels × 1,024 pixels) at distances of 2 to 3 μm in the axial direction. The pinhole size was 1 airy unit. In some cases the thickness of the tissue did not allow to capture a complete image stack of a good quality, therefore the preparation was turned upside down and scanned from the opposite side to achieve a stronger emitted signal. The image material was further edited using Adobe Photoshop CS6 for brightness and contrast adjustment. Adobe Illustrator CS6 was used for the final panel composition.

### Digital Reconstruction and Registration of Neurons into the Standard Brain Model

In order to design 3D images and spatial relationship of neurons from raw data, the Amira 5.3 (Visualization Science Group) software was used. The neuropils of interest were labeled manually by using the segmentation editor. To trace neuronal filaments and to reproduce axon thicknesses and exact positions, the skeletonize plugin for Amira5.3 (Schmitt et al., 2004; Evers et al., 2005) was used. To compensate for refraction indexes, the *z*-axis dimension of the brain was multiplied by a factor of 1.16 for the water objective and 1.54 for the air lens. Registration of the skeleton tree into the standard model was described in Kvello et al. (2009). The segmented images of the specimen were affine and elastically registered to the corresponding label images of the standard model. The affine transformation matrixes and the deformation vector fields were subsequently applied to the reconstructed skeleton tree, and by this the neuron was registered into the standard brain.

Some neurons were reconstructed without being registered into the standard brain atlas of the male. This ‘non-integrated’ class includes PNs confined to brain preparations that were not sufficiently stained by the synapsin antibody, thus making it difficult to reconstruct all the relevant brain structures. Each of these neuron models is presented in a simplified reconstruction of the particular brain they originated from. Here, no other structures than the antennal lobe, the central body, and the Ca are indicated. Two female PNs passing in the tALT and the lALT, respectively, were registered into the standard brain atlas of the *H. virescens* female (Kvello et al., 2009).

For defining neuropils not included in the standard brain atlas, but targeted by the various PN types, we utilized confocal scans of brain preparations labeled via synapsin immunostaining and made indicative reconstructions of the relevant areas. So-called unspecified neuropil regions have previously been characterized in the lepidopteran brain by Heinze and Reppert (2012). The reconstructions, which were made by using the segmentation tool in AMIRA, were manually transferred into the standard brain model. Detailed information regarding definition of the distinct neuropil regions is stated as online Supplementary Material.

### Nomenclature

For naming the neuropil structures of the brain, we used the nomenclature newly established by Ito et al. (2014). However, as concerns definition of the LH, we have restricted this region in the moth brain to include the area targeted by the uni-glomerular PNs, plus its adjacent zones. Defining the LH as the target region of *all* antennal-lobe PNs, as stated in Ito et al. (2014), makes no sense in moths. One main reason is that a prominent sub-branch of the lALT projects to a region located in the medial protocerebrum. For naming the sub-classes of PNs confined to the various ALTs, we have used the system established by Homberg et al. (1988), however, adjusted to the new names of the tracts. The orientation of all brain structures is indicated relative to the body axis of the insect, as in Homberg et al. (1988).

## Results

In total, our results include 46 labeled PNs, each confined to one of five ALTs - the medial, the medio-lateral, the lateral, the transverse, or the dorso-medial ALT (**Table 1**). Morphologically different sub-types of PNs were found within all the three classical ALTs, i.e., the mALT, the lALT, and the mlALT. As previously mentioned, neurons were categorized in accordance with the system used by Homberg et al. (1988) except for the reference to the relevant tract, which was changed corresponding to the new nomenclature by Ito et al. (2014), meaning that the uniglomerular PNs confined to the mALT, for example, named PIa by Homberg et al. (1988), are named Pm_a here.

**Table 1:** Total overview of labeled projection neurons (PNs), each confined to one of five antennal-lobe tracts.

ALTs	Cell body	Glomerular arborization	Terminal projections	Number of PNs
**mALT**				**Total: 32**
Pm_a	LC	UG	Ca; LH	29
Pm_d	LC	UG	Ca; LH; INP; AVLP	1
Pm_e	ACC	MG	Ca; LH; INP; SNP; VMNP; AVLP	1
Pm_f	LC	MG	VMNP; PLP	1
**lALT**				**Total: 6**
Pl_a_uni	LC	MG	Ipsilateral column	2
Pl_a_bi	LC	MG	Ipsilateral and contralateral columns	1
Pl_b	LC	MG	AVLP	1
Pl_e	LC	MG	PVLP; SNP	1
Pl_f	LC	MG	LH; INP	1
**mlALT**				**Total: 6**
Pml_a	LC	MG	LH	4
Pml_b	LC	MG	LH; SNP; PLP; the base of the Ca	2
**tALT**				**Total: 1**
Pt	LC	MG	LH; INP; PVLP; GNG	1
**dmALT**				**Total: 1**
Pdm	GNG	UG	Ca; LH	1


*Ca, calyx; LH, lateral horn; VMNP, ventro-medial neuropil; INP, inferior neuropil; PLP, posterior- lateral protocerebrum; AVLP, anterior ventro-lateral protocerebrum; SNP, superior neuropil; PVLP, posterior ventro-lateral protocerebrum; GNG, gnathal ganglion.*

### Projection Neurons Confined to the mALT: Pm Neurons

Altogether, 32 PNs passing along the prominent mALT were stained (**Table 1**). In correspondence with previous reports, the most frequently encountered type of medial-tract neurons was found to be the classical uniglomerular neuron with sequential arborizations in the Ca and the LH (Homberg et al., 1988; Rø et al., 2007). In addition to this type (Pm_a neurons), we present three morphologically novel medial-tract PNs, named Pm_d, Pm_e, and Pm_f. We found no neurons corresponding to the PIb and PIc types reported by Homberg et al. (1988).

#### Pm_a Type

**FIGURE 1:**
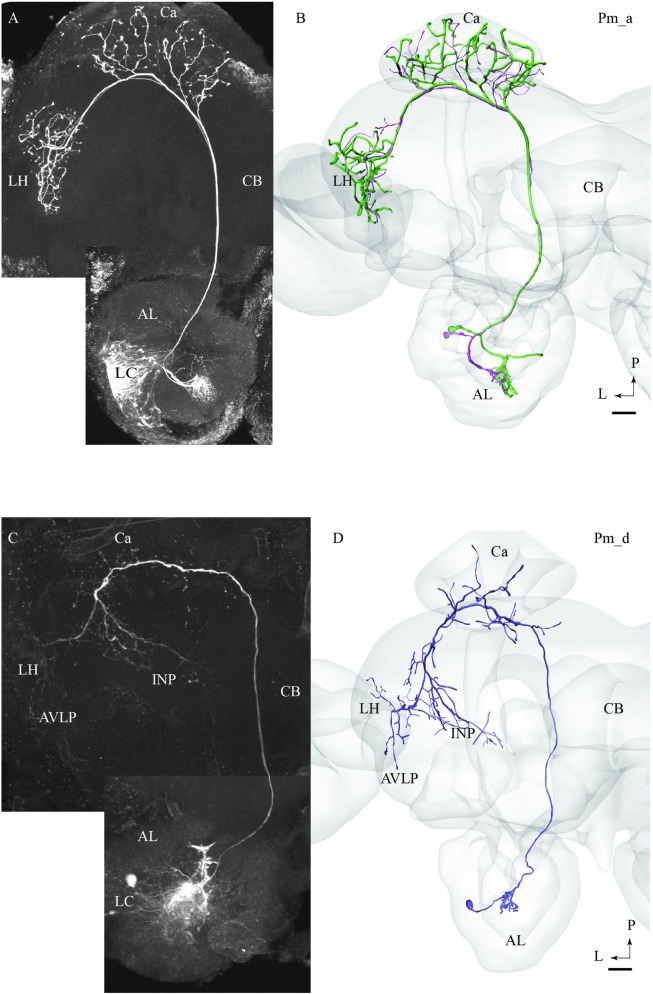
**Two morphological sub-types of uniglomerular medial-tract neurons, one classical **(A,B)** and one non-classical **(C,D)**.** **(A)** Confocal image of two simultaneously stained uniglomerular medial-tract neurons originating in the same glomerulus. The two ‘sister’ Pm_a neurons project to overlapping regions in the Calyx (Ca) and the lateral horn (LH), and their cell bodies are located adjacently in the lateral cell cluster (LC). **(B)** 3D reconstruction of the two Pm_a neurons presented in **(A)**, registered into the standard brain atlas. **(C)** Confocal image of a uniglomerular medial-tract neuron of type Pm_d. **(D)** 3D reconstruction of the Pm_d neuron presented in **(C)**, registered into the standard brain atlas. In addition to the Ca and the LH, this neuron targets the anterior ventro-lateral protocerebrum (AVLP) and the inferior neuropil (INP). The cell body is located in the LC. CB, central body; P, posterior; L, lateral. Scale bars = 50 μm.

Most of the stained neurons confined to the mALT belonged to the category of uniglomerular PNs innervating the Ca before terminating in the LH (named PIa neurons in Homberg et al., 1988). Actually, 29 of the 32 medial-tract PNs stained were categorized as type Pm_a. Generally, Pm_a neurons innervating dorso-medially situated glomeruli in the AL were found to pass along the dorsal root, being linked to the medial cell body cluster, whereas neurons confined to more ventro-lateral glomeruli passed along the ventral root, which is connected to the lateral cell body cluster (LC). One typical example of Pm_a neurons is shown in **Figures 1A,B**; here, two sister neurons innervating the same glomerulus can be seen. Except for a difference in the thickness of the neural processes, the two neurons appear very similar, including cell bodies located adjacently in the LC and terminal branches showing complete overlap not only in the Ca but also in the LH. The only distinction in projection pattern was a short side branch to a neighboring AL glomerulus extending from the thickest PN. All Pm_a neurons displayed a projection pattern similar to that characterizing the two sister neurons described above.

#### Pm_d Type

One of the stained medial-tract PNs resembled the classical uniglomerular type by arborizing in one AL glomerulus and innervating the Ca before terminating in the ipsilateral protocerebrum. However, the protocerebral projections of the Pm_d type covered a substantially wider area. As shown in **Figures 1C,D**, the main axon of this neuron extended one medially directed sub-branch on its route from the Ca to the LH, terminating in the inferior neuropil. In its lateral continuation, the main axon innervated the LH and the anterior ventro-lateral protocerebrum, which constitutes a part of the ventro-lateral neuropil (**Figures 1C,D**). Another distinctive feature of this neuron was its sparse innervations – only a few thin branches – within the Ca. The neuron had its cell body located in the LC.

#### Pm_e Type

**FIGURE 2:**
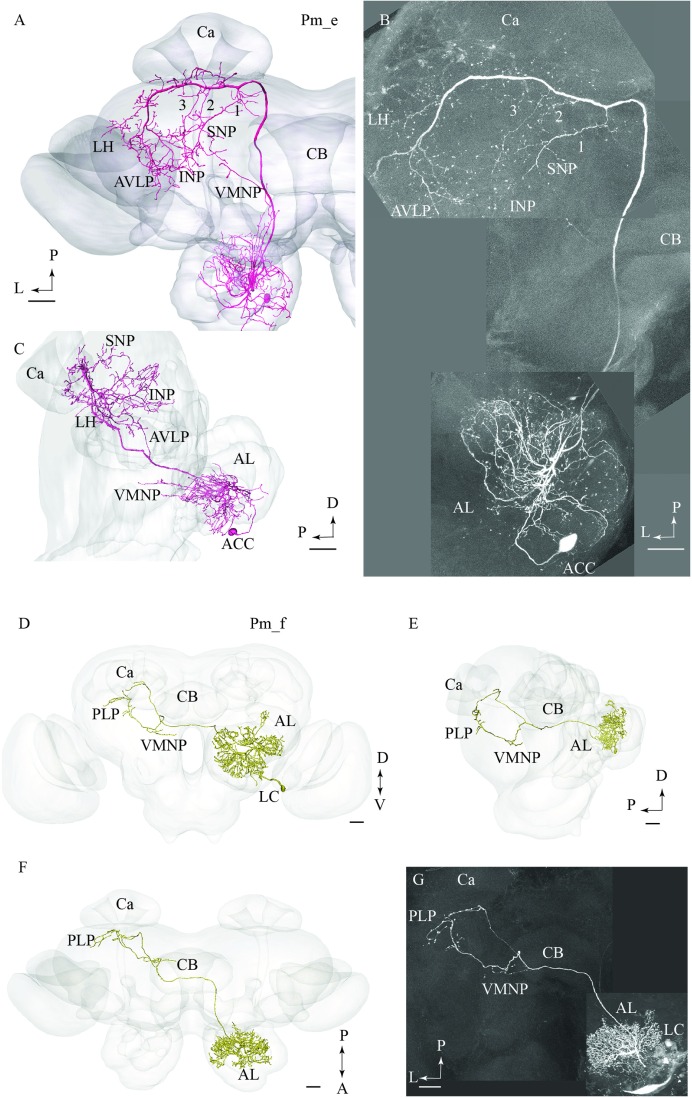
**Two untypical medial-tract neurons, presented by means of confocal images and 3D-reconstructions registered into the standard brain atlas.** **(A–C)** One neuron, named Pm_e, displays multiglomerular ramifications in the antennal lobe (AL) and targets wide-spread terminal areas in the ipsilateral protocerebrum. In the AL, the neuron innervates the periphery of several glomeruli located in the postero-ventral region. The relatively large cell body is located in the anterior cell body cluster (ACC). In the protocerebrum, the main axon extends numerous short processes along its entire lateral course toward the LH. Three anteriorly directed sub-branches, numbered 1, 2, and 3, split off from this fragment of the axon. Together, the continuation of the main axon and these collaterals target the INP, the superior neuropil (SNP), the LH, and the AVLP. A small region in the ventro-medial neuropil (VMNP) is innervated by a small sub-branch that splits off from the axon close to the ventro-lateral edge of the central body (CB). Only a few processes can be seen in the Ca (Ca). **(D–G)** A contralateral medial-tract neuron, named Pm_f, ramifies densely in the posterior region of one AL, forming a non-glomerular pattern. The cell body is located in the lateral cell body cluster (LC). The axon passes along the path of the medial antennal-lobe tract until it reaches the CB. Here, it bends off and projects to the contralateral hemisphere terminating in the ventro-medial neuropil (VMNP) and the posterior lateral protocerebrum (PLP). D, dorsal; V ventral; P, posterior; A, anterior. Scale bars = 50 μm.

The type named Pm_e included one medial-tract PN displaying an entirely unique branching pattern compared to the common category (**Figures 2A,B**). This neuron had its soma located in the smallest cell body group in the AL, the anterior cell cluster, and thus belongs to a population of PNs rarely described previously. As shown in **Figures 2A,B**, the Pm_e neuron arborized in numerous glomeruli located ventrally, posteriorly, and medially in the AL. Unlike the uniglomerular PNs, which innervate the glomerular core, this neuron extended smooth processes along the periphery of the individual glomeruli enclosing each spherical unit. All in all, this forms a characteristic loop-like pattern in the AL. The cell body was relatively large (17 μm), and the primary neurite passed through the AL outside the two main roots. The main axon projected posteriorly along the course of the medial tract, and immediately after turning laterally in the posterior protocerebrum, it extended three consecutive anterior-laterally directed sub-branches (**Figures 2A,B**). The first branch, positioned most medially, projected dorsally and terminated in the superior neuropil whereas the two remaining branches targeted the region adjacent to the peduncle before terminating in the inferior neuropil. Also, the main axon sent off short neural branches along its lateral course anteriorly of the Ca. These terminals might be located adjacent to one of the superior commissures. The innervation of the Ca was scarce; only a few thin processes targeted the most basal part of this neuropil. The main axon passed on via the lateral continuation of the mALT and innervated a relatively large region of the lateral protocerebrum including the LH and the anterior ventro-lateral protocebrum. Also, one small side branch extending from the mALT at the edge of the central body terminated in the medial part of the inferior neuropil. Finally, a few thin processes originating from the posterior part of the AL projected directly into an anterior region of the ventro-medial neuropil (**Figure 2C**).

#### Pm_f Type

The fourth type of medial-tract PN was a contralateral neuron. As demonstrated in **Figures 2D–G**, the axon of this Pm_f neuron projected from the AL via the initial part of the ipsilateral mALT, but then split off this path and continued along the ventral border of the central body to the contralateral protocerebrum. The neuron possessed dense arborizations covering a substantial portion of the AL including the posterior region. Its ramifications did not form a typical glomerular pattern. The cell body was located in the LC. In the contralateral hemisphere, the neuron split into two sub-branches ventrally and posteriorly of the central body. Here, the first branch targeted the ventro-medial neuropil before projecting further and terminating in the posterior lateral protocerebrum in close proximity to the pedunculus. The second sub-branch projected dorso-posteriorly toward the base of the Ca terminating adjacently to one of the terminal endings of the first sub-branch.

### Projection Neurons Confined to the lALT: Pl Neurons

In total, six PNs passing along lALT were labeled (**Table 1**). As compared to the medial-tract neurons, consisting of a major group of uniform neurons, the PNs confined to the lALT formed a generally heterogeneous group. Except for having their cell body located in the LC and sharing the initial route, projecting dorso-laterally from the AL, these neurons were morphologically different in that they proceeded further along distinct trajectories terminating in various protocerebral regions. None of the lateral-tract PNs presented here innervated the Ca.

#### Pl_a_uni Type

**FIGURE 3:**
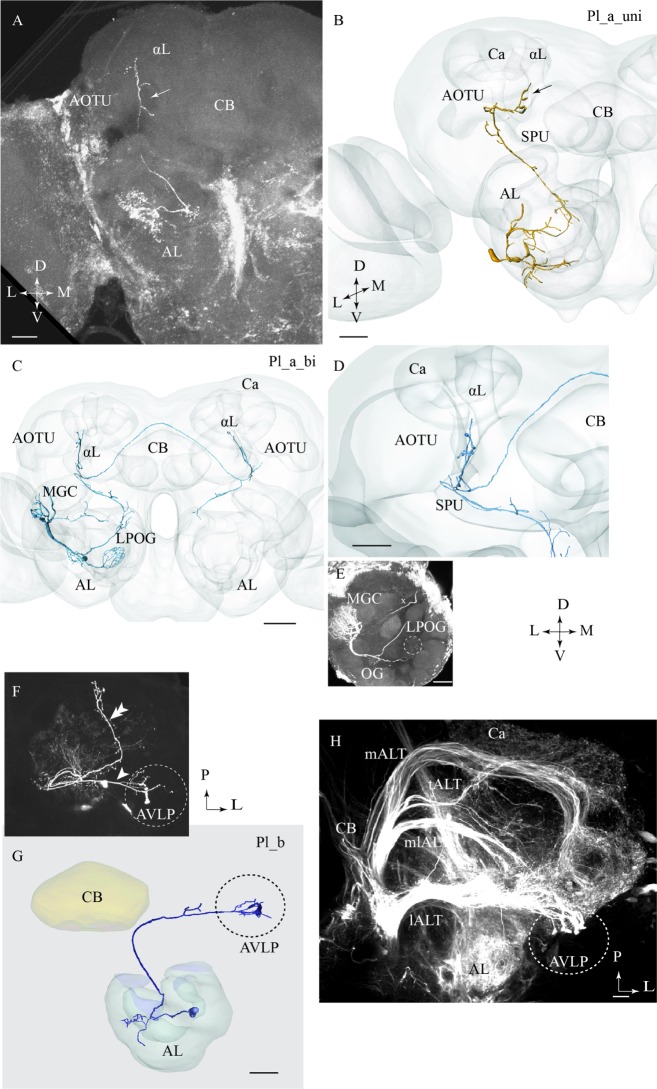
**Three different sub-categories of lateral-tract neurons, named Pl_a_uni, Pl_a_bi, and Pl_b, presented by means of confocal images and 3D reconstructions.** **(A)** and **(B)** The Pl_a_uni neuron follows the initial dorso-lateral course of the lateral antennal-lobe tract, but then, at the spur (SPU) of the mushroom bodies, it bends off from this path and continues dorsally terminating in a region between the anterior optic tubercle (AOTU) and the alpha-lobe (αL), the so-called column (arrow). **(C–E)** The Pl_a_bi neuron displays a projection pattern comparable to that of the aforementioned neuron by terminating in the column. This bilateral neuron sends off a projection to the contralateral hemisphere, targeting a region symmetrical to that described above. Among the glomeruli innervated in the ipsilateral AL, is the cumulus of the macro glomerular complex (MGC) and the labial pit organ glomerulus (LPOG). **(F–H)** The third neuron category, named Pl_b, projects directly to the AVLP. Here, it extends a few relatively short processes, one of which ends up in a prominent club-like structure. The Pl_b neuron in **(F),** indicated by an arrowhead, was stained simultaneously with a Pl_a_uni neuron (double arrowhead). The 3D reconstruction in **(G)** presents the distinct morphology of the individual Pl_b neuron. The confocal image in **(H)** shows mass-stained antennal-lobe projection neurons (PNs) including a population of approximately 10 lateral-tract neurons possessing a characteristic terminal ending in the AVLP, identical to the club-like structure mentioned above (indicated by the dashed circle). Ca, calyces; L, lateral; P, posterior; M, medial; A, anterior. Scale bars = 50 μm.

Two of the stained lateral-tract neurons were characterized by an unbranched terminal projection forming a pillar-like structure in the superior intermediate protocerebrum between the AOTU and the alpha-lobe. We have named this region the *column*. The Pl_a_uni neurons (uni for unilateral) did not target the LH, but turned off from the initial course of the lALT at the spur of the mushroom bodies and projected dorsally to the column. These PNs, which correspond to the POa neurons in Homberg et al. (1988) and Rø et al. (2007), extended small bleb-like protrusions along their entire route (**Figures 3A,B**). Both neurons arborized in several AL glomeruli.

#### Pl_a_bi Type

One lateral-tract neuron, categorized as Pl_a_bi, was comparable with the aforementioned type in that it targeted the demarcated area between the AOTU and the alpha-lobe, i.e., the column. This PN was a bilateral neuron, however. As shown in **Figures 3C,D**, the neuron’s axon turned off from the main lALT course at the spur of the mushroom bodies. At the turning point, the neuron extended, in addition to the projection terminating the column, an axon passing along the posterior border of the central body into the contralateral protocerebrum. Here, in the other hemisphere, the axon further split in two sub-branches, one innervating the column and the other targeting the AL. In the ipsilateral AL, this neuron innervated three distinct glomeruli: the cumulus of the MGC, the labial pit organ glomerulus (LPOG), and one ordinary glomerulus (**Figure 3E**). A fine connection between the cumulus and some ordinary glomeruli located centrally in the AL could also be observed. The weak staining in the contralateral AL did not allow us to reconstruct the entire axonal arborizations. Generally, however, the neuron displayed a bilaterally symmetric projection pattern (**Figure 3C**).

#### Pl_b Type

One lateral-tract PN extended a few short terminal branches in the anterior ventro-lateral protocerebrum, one of which ended in a prominent club-like structure (**Figures 3F,G**). This Pl_b neuron, which was labeled simultaneously with another lateral-tract neuron of type Pl_a, corresponds to a group previously classified as POb by Homberg et al. (1988). Confocal data from mass staining experiments in our lab have demonstrated a small assembly of club-like terminals in the same region of the anterior ventro-lateral protocerebrum, indicating the presence of approximately 10 PNs displaying morphological properties similar to the type presented here (**Figure 3H**). The confocal images allowed us to make a separate reconstruction of the stained Pl_b neuron (**Figure 3G**) and here we show the complete morphology of this neuron type.

#### Pl_e Type

**FIGURE 4:**
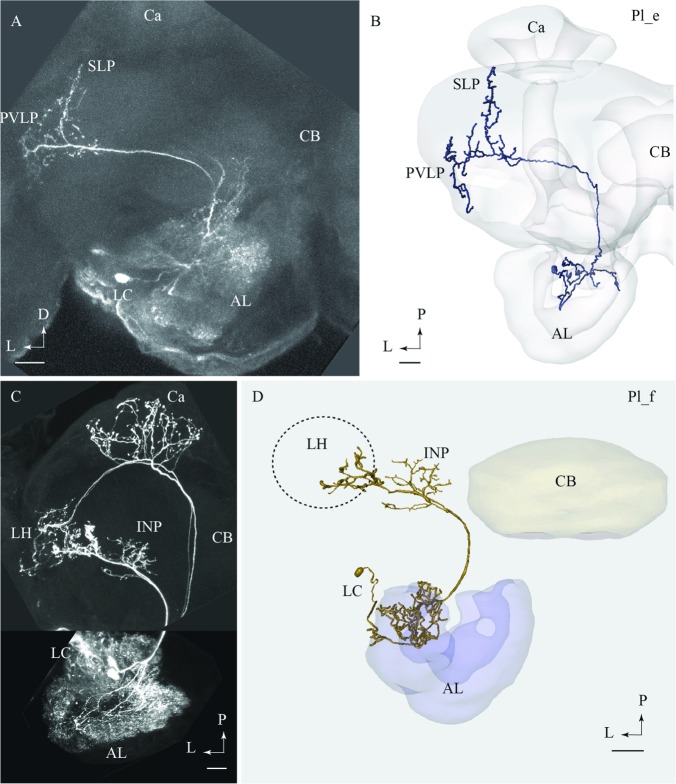
**Two lateral-tract neurons classified as distinct types, Pl_e and Pl_f, respectively.** **(A)** and **(B)** Confocal image **(A)** and 3D reconstruction **(B)** of the Pl_e neuron demonstrating its multiglomerular arborizations in the AL and location of cell body in the lateral cell body cluster (LC), plus its projections to the SLP and posterior ventro-lateral protocerebrum (PVLP). **(C)** and **(D)** Confocal image and 3D reconstruction of the lateral-tract neuron classified as Pl_f. The Pl_f neuron was stained together with a classical medial-tract neuron. The 3D reconstruction of the Pl_f neuron demonstrates its multiglomerular arborizations in the AL and axonal projection terminating in the INP and the ventral part of the LH. Ca, calyces; CB, central body; P, posterior; L, lateral. Scale bars = 50 μm.

One multiglomerular lALT neuron, classified as Pl_e type, had its cell body located in the LC and terminal branches in the lateral protocerebrum, but not in the LH. On its lateral path, the axon extended a side branch projecting dorso-posteriorly (**Figures 4A,B**). This collateral, which sent off short processes along its course, terminated in the superior lateral protocerebum, nearby the Ca. The continuation of the main projection terminated in the posterior ventro-lateral protocerebrum without innervating the LH. In the AL, the Pl_e neuron arborized in the most ventrally located glomeruli. This PN was obtained from a female and is therefore registered into the female standard brain atlas.

#### Pl_f Type

Among all stained PNs confined to the lALT, only the Pl_f terminated in the LH. On its route to the LH, this neuron extended terminal branches innervating the inferior neuropil (**Figures 4C,D**). The neuron had multiglomerular ramifications in the AL and its cell body was positioned in the LC. This lateral-tract neuron was stained simultaneously with two PNs confined to the mALT.

### Projection Neurons Confined to the mlALT: Pml Neurons

**FIGURE 5:**
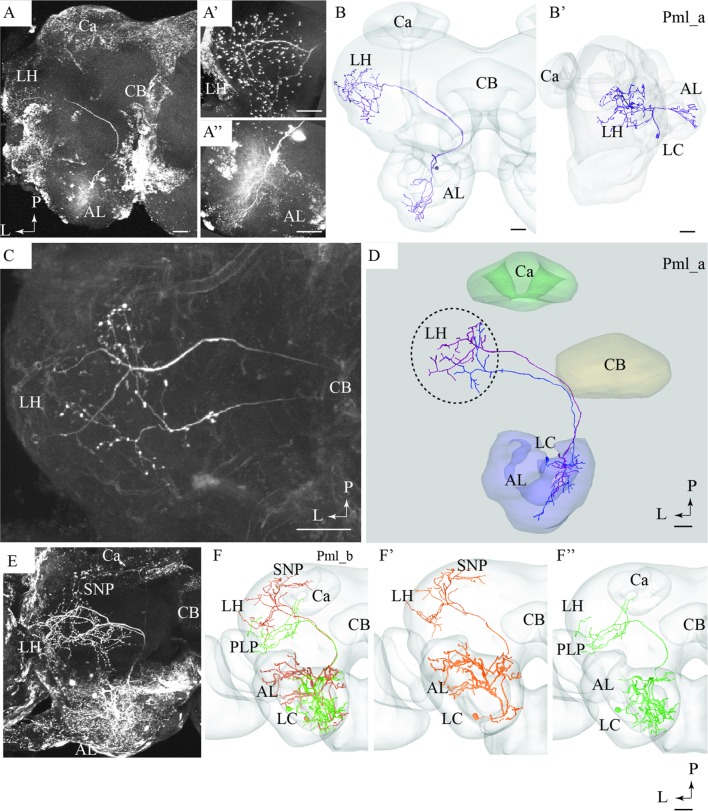
**Confocal images and 3D reconstructions of the two neuron types confined to the medio-lateral antennal-lobe tract.** **(A–D)** Three neurons of the Pml_a type, targeting the lateral horn (LH) only: one neuron in **(A,B)** and two, simultaneously stained, in **(C,D)**. The direct connection to the LH, the widely spread arborizations and bouton terminals in the LH **(A’)**, and the multiglomerular innervations in the AL are shown **(A”)**. **(E)** and **(F)** Two Pml_b type neurons labeled simultaneously. The two Pml_b neurons innervate different protocerebral regions in addition to targeting overlapping terminal regions in the LH. Their cell bodies are located in the lateral cell body cluster (LC). **(F’,F”)** Separated 3D reconstructions of the two Pml_b neurons showing their individual morphologies: one projects to the superior neuropil (SNP) and the other to the PLP. Ca, calyces; CB, central body; P, posterior; L, lateral. Scale bars = 50 μm.

All six mlALT PNs stained were multiglomerular and had cell bodies in the LC (**Figure 5**). Based on their projection pattern in the protocerebrum, we classified these mlALT PNs into two groups.

#### Pml_a Type

A typical mlALT PN, named Pml_a, is exemplified in **Figures 5A,B**. This neuron split off the mALT at the edge of the central body and ended in a relatively large area within the LH. Its terminal branches possessed numerous blebs distributed across the entire target region (**Figure 5A**’). Two similar mlALT neurons, stained simultaneously, are presented in **Figures 5C,D**. As demonstrated, these two neurons innervated partly different areas within the LH. One additional Pml_a neuron was identified, but not presented in the figures.

#### Pml_b Type

The two remaining mlALT neurons which were also stained simultaneously showed a slightly different morphology by innervating distinct regions in addition to the LH (**Figures 5E,F**). The confocal image of this preparation allowed individual identification of the two neurons and each could thus be reconstructed separately. As shown in the AMIRA models, these neurons terminated in partly different regions of the protocerebrum (**Figure 5F**). One neuron targeted the LH and the superior neuropil whereas the second neuron sent sub-branches to the posterior lateral protocerebrum and the base of the Ca, in addition to the LH, (**Figures 5F**’,F”, respectively).

### Projection Neurons Confined to the tALT and dmALT, Respectively: Pt and Pdm Neurons

**FIGURE 6:**
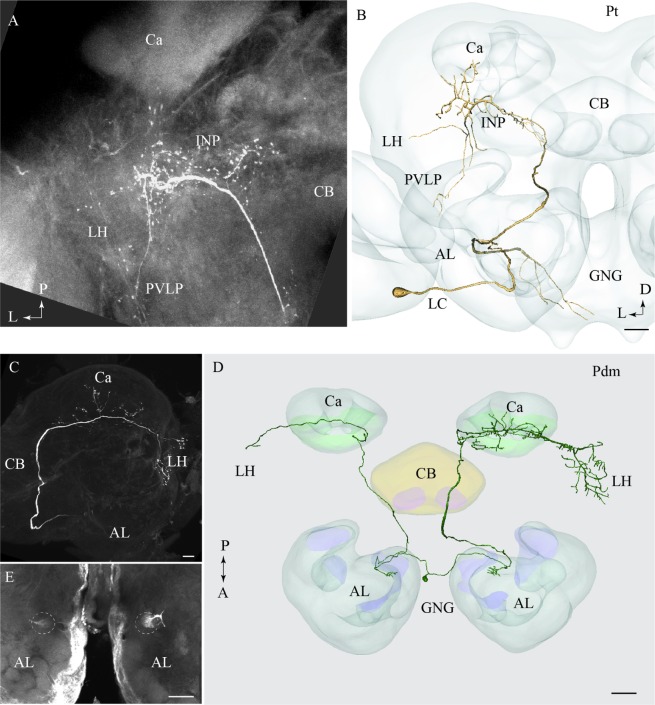
**Two individual neurons confined to the transverse and the dorso-medial antennal-lobe tract, respectively.** **(A)** and **(B)** Confocal image and 3D reconstruction of the transverse-tract neuron, named Pt. The cell body is located in the lateral cell body cluster (LC) and the neuron extends only a few thin branches in the AL. The main axon passes along the course of the medial antennal-lobe tract (mALT) until it turns dorso-laterally adjacent to the CB and terminates in a region of the INP surrounding the pedunculus. Also, a few branches extend to the base of the Ca, the LH, and the PVLP. In addition to the main axon passing along the initial path of the mALT, a second fiber terminating in the gnathal ganglion (GNG) leaves the AL. **(C–E)** Images of a bilateral neuron confined to the dorso-medial antennal-lobe tract (dmALT), named Pdm. The confocal image in C shows the general projection pattern in the left hemisphere including terminal branches innervating the calyces (Ca), and the LH. The 3D reconstruction in D displays the whole Pdm neuron including its two symmetrically located glomeruli of the ALs, each of which is innervated by one fiber running from the cell body that is located in the GNG. The confocal image in E visualizes the two symmetric glomeruli innervated by the Pdm neuron. CB, central body; P, posterior; A, anterior. Scale bars = 50 μm.

One neuron projecting in the tALT was stained (**Figures 6A,B**). This Pt neuron, which had its cell body located in the LC, showed a sparse innervation pattern within the AL. The main axon left the AL along the path of the mALT, but turned off from the posterior direction and projected laterally close to the edge of the central body, posteriorly of the split-point for the mlALT. The neuron extended numerous short processes within the inferior neuropil (**Figure 6B**). Additional branches sparsely innervated distinct areas of the LH, the posterior ventro-lateral protocerebrum, and the base of the Ca. This neuron also extended one prominent axon projecting from the AL to the gnathal ganglion (GNG; **Figure 6B**). The Pt neuron was obtained from a female and is therefore registered into the female standard brain atlas.

One bilateral neuron projecting in the dmALT was stained (**Figures 6C–E**). Its cell body was located in the GNG, close to the midline. This Pdm neuron innervated both ALs showing arborizations in two symmetrically positioned glomeruli located ventro-medially (**Figure 6E**). From the AL, an axon projected along the path of the dmALT in each hemisphere. Thus, the two axons passed in a posterior direction, relatively close to the brain midline. In contrast to the medial-tract PNs, this neuron passed dorsally of the central body. The neuron targeted the Ca and the LH. Weak staining of the right brain hemisphere did not allow full reconstruction of the neuron. However, the raw data allow us to assume that the neuron innervated symmetrically positioned neuropils in the protocerebrum as well, probably with more extensive projections in one hemisphere.

**FIGURE 7:**
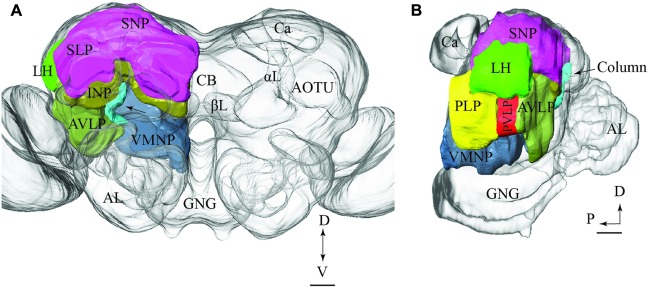
**Model brain with indication of the main regions targeted by PNs confined to the various antennal-lobe tracts (the reconstructions are limited to the regions innervated by the stained neurons).** **(A)** Frontal view (tilted slightly dorsally). The arrow points to the column. **(B)** Sagittal view. AL, antennal lobe; GNG, gnathal ganglion; αL, alpha lobe; βL, beta lobe, AOTU, anterior optic tubercle; Ca, calyces; LH, lateral horn; AVLP, anterior ventrolateral protocerebrum; PVLP, posterior ventrolateral protocerebrum; PLP, posterior lateral protocerebrum; VMNP, ventromedial neuropil; SNP, superior neuropil; SLP, superior lateral protocerebrum (of the SNP); INP, inferior neuropil; CB, central body. Scale bars: 50 μm.

To visualize the output areas of all PNs presented here, we have utilized the confocal data by labeling the relevant neuropil areas and manually transformed these data into the standard brain model (**Figure 7**).

### Integration of Individual Neuron Models into the Standard Brain Atlas

The possibility of registering neuron models originating from different individuals into the standard brain atlas allowed for comparing the projection patterns of several PNs, including those passing in the same ALT as well as neurons confined to different tracts.

#### PNs Confined to the mALT

**FIGURE 8:**
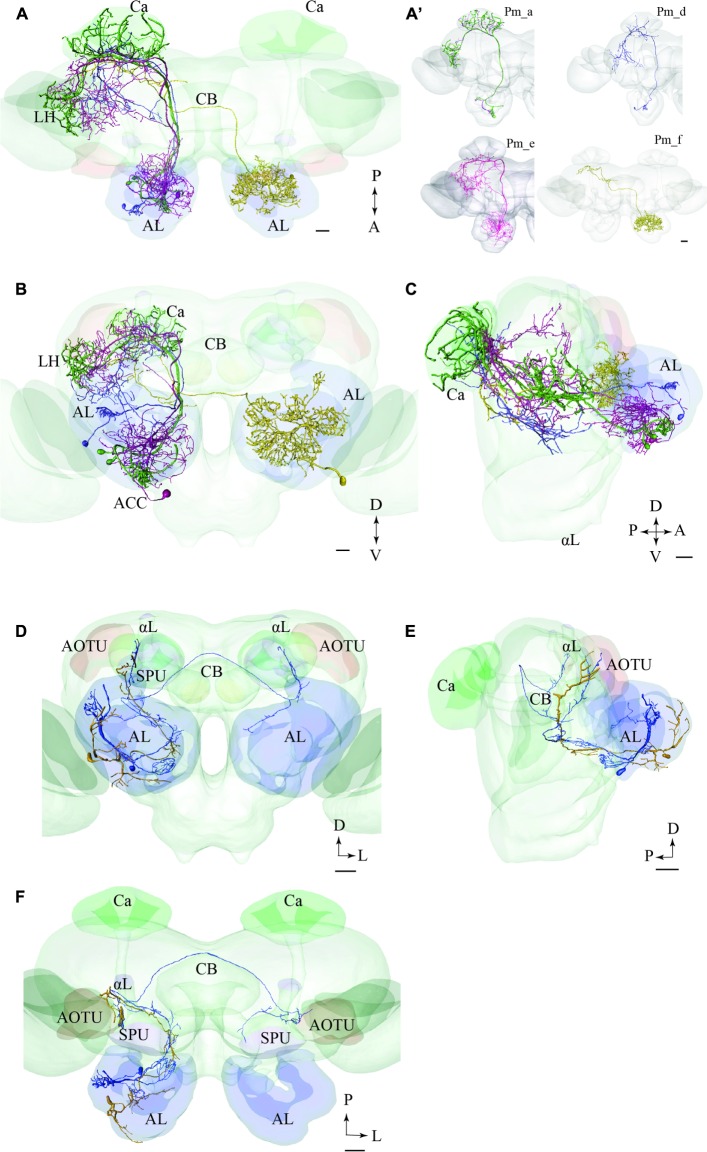
**Two examples showing integration of registered neurons into the standard atlas, both including neurons confined to the same tract.** **(A–C)** Image of four neuron types confined to the medial tract in a dorsal **(A)**, frontal **(B)**, and sagittal **(C)** orientation, respectively, demonstrating their widespread protocerebral target areas. Except for the contralateral neuron (yellow) all Pm types show partial overlap in the LH. **(A’)** Overview of the four individual Pm neuron types, named Pm_a, Pm_d, Pm_e, and Pm_f. **(D–F)** Two lateral-tract PNs, one bilateral (Pl_a_bi) and one ipsilateral (Pl_a_uni) showing similar projections along the initial trajectory and terminal output in the column located between the AOTU and the alpha lobe (αL). AL, antennal lobe; Ca, calyces; CB, central body; ACC, anterior cell body cluster; P, posterior; A, anterior; D, dorsal; V, ventral. Scale bars = 50 μm.

In **Figures 8A–C**, we present the various types of medial-tract PNs. As demonstrated, the classical uniglomerular type, Pm_a (shown in green), targets the Ca and the LH whereas the other types, including Pm_d (blue), Pm_e (magenta), and Pm_f (yellow), target mainly other protocerebral regions. Even though Pm_d (blue) and Pm_e (magenta) overlap partly with the Pm_a type (green) in the LH, most of the terminal branches of these neurons cover more medially located regions in the superior, inferior, and ventral protocerebrum. The contralateral PN, Pm_f (yellow), targets a restricted area nearby the base of the pedunculus and thus terminates in a region also innervated by the Pm_e neuron (magenta).

#### PNs Confined to the lALT

In **Figures 8D–F**, we present two individual PNs belonging to different categories of latera-tract neurons (Pl_a_uni and Pl_a_bi). A prominent feature characterizing these neuron types is their projection terminating between the AOTU and the alpha-lobe of the mushroom body, in the pillar-shaped region termed the column.

#### PNs Confined to the mALT and mlALT, Respectively

**FIGURE 9:**
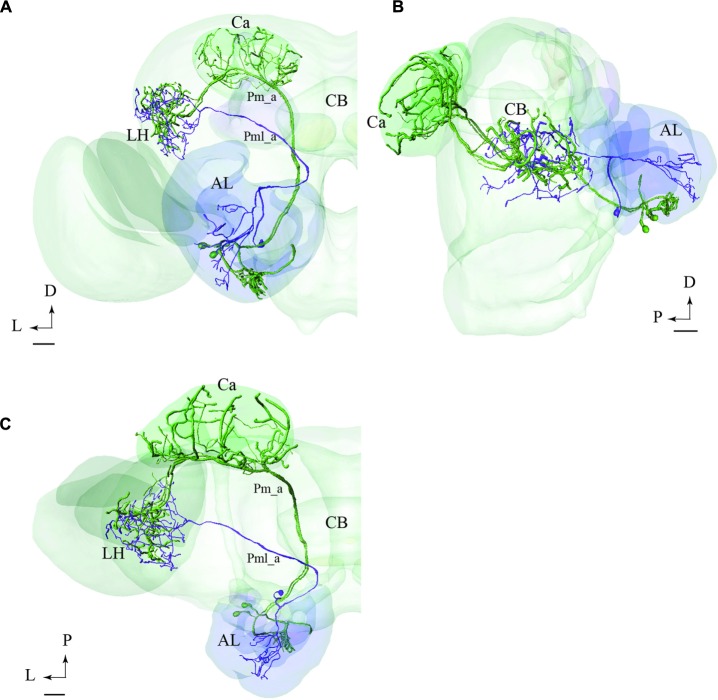
**Projection neurons confined to two different tracts, the mALT and the mediolateral antennal-lobe tract (mlALT): Registration of the Pm_a type neurons (green) and the Pml_a neuron (blue) into the standard brain atlas demonstrates that the neurons project to the same area in the LH.** **(A–C)** Frontal, sagittal, and dorsal view, respectively. AL, antennal lobe; Ca, calyces; CB, central body; D, dorsal; L, lateral; P, posterior. Scale bars = 50 μm.

In **Figure 9**, we have integrated models of PNs passing along two different tracts, i.e., the medial and the mediolateral. Here we present the main neuron category confined to each of the two tracts, i.e., the Pm_a and Pml_a type. As demonstrated, there is a considerable overlap of terminal regions in the LH.

### Comparison of LH Terminal Areas of mALT and lALT Neurons

**FIGURE 10:**
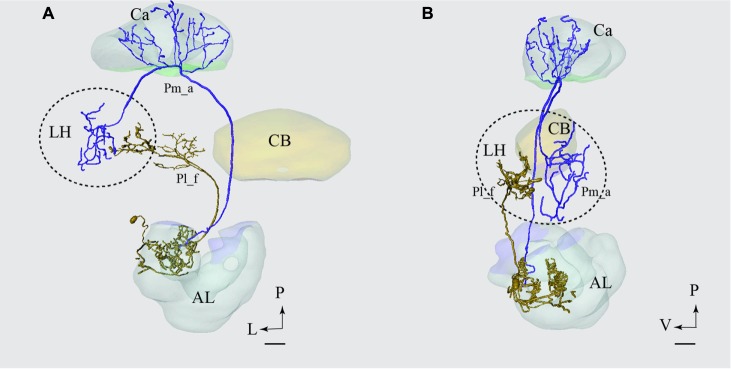
**Projection neurons confined to two different tracts, the mALT and the lateral antennal lobe tract (lALT), terminating in different regions.** Reconstruction of the two simultaneously stained PNs, the medial-tract neuron (Pm_a) in blue and the lateral-tract neuron (Pl_f) in yellow, shows that both neurons send terminal branches in the LH, but to different areas. The dorsal **(A)** and the sagittal **(B)** view of the two reconstructed neurons demonstrate that the Pm_a neuron targets a lateral-horn area located more laterally and dorsally than that innervated by the Pl_f neuron. AL, antennal lobe; Ca, calyces; CB, central body; P, posterior; L, lateral; V, ventral. Scale bars = 50 μm.

The only type of the lateral-tract neurons that terminated within the LH was Pl_f type. To compare its target area in the LH with that of the medial-tract neurons, we used the individual reconstruction of Pm_a and Pl_f neurons that were simultaneously labeled within the same preparation (**Figure 4C**). As demonstrated in **Figure 10**, their terminal endings in the LH show no overlap; the lateral-tract neuron projects to a region located more ventrally and medially than that innervated by the medial-tract neuron.

## Discussion

**FIGURE 11:**
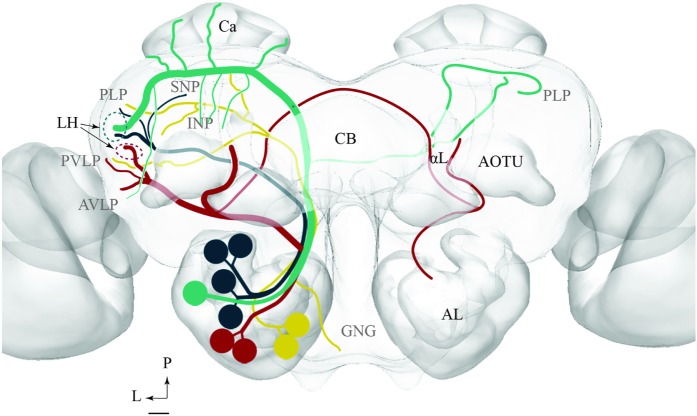
**Schematic drawing showing the projection patterns of various antennal-lobe output neurons confined to the medial (green), mediolateral (dark green), lateral (red), and transvers (yellow) antennal lobe tracts.** The dorsomedial tract is not included. The thin sub-branches indicate un-typical target regions such as the SNP, PLP, PVLP, and AVLP. The majority of neurons projecting in the medial and the mediolateral ALTs target an overlapping region in the lateral horn (LH; green circle), whereas neurons confined to the lateral antennal-lobe tract terminate in a more ventral part of the LH (red circle). AL, antennal lobe; CB, central body; Ca, Calyces; AOTU, anterior optic tubercle; GNG, gnathal ganglion; INP, inferior neuropil; αL, alpha lobe; P, posterior; L, lateral. Scale bar: 50 μm.

The results presented here contribute to the anatomical analysis of the moth AL pathways elucidating the morphological characteristics of PNs both across and within the ALTs. Altogether 46 PNs belonging to five ALTs were morphologically identified: 32 in the mALT, six in the lALT, six in the mlALT, one in the tALT, and one in the dmALT (**Table 1**). The classification of PNs within each tract was performed according to their terminal outputs in different protocerebral areas. Further, the digital reconstruction and registration into the standard brain gave us the opportunity to visualize individual PNs and to compare output areas of the different neurons both across and within distinct tracts (**Figure 11**).

### Morphological Properties of PNs Confined to the Different ALTs

#### Projection Neurons Passing in the mALT

The present study together with previous findings obtained from other insect orders including cockroaches, beetles, flies, bees, and ants (Galizia and Rössler, 2010; Martin et al., 2011) demonstrate that the “classical” uniglomerular PNs constitute the absolutely most prominent portion of the mALT (in our study 29 neurons out of 32). The main morphological feature of these second order neurons is their terminal projections exclusively to the Ca and the LH. The simultaneous staining of two Pm_a neurons arborizing in the same AL glomerulus, as shown in **Figures 1A,B**, enabled inspection of their projection patterns in the higher brain regions; the complete overlap of their axon terminals in the LH agrees with previous data from *Drosophila* showing that PNs originating from the same glomerulus project to one distinct area in the LH (Yu et al., 2010; Tanaka et al., 2012). Recently, male-specific third order olfactory neurons were described in *B. mori*. In addition to the LH, the complete pheromone circuit comprises the superior medial protocerebrum and the lateral accessory lobe (Namiki et al., 2015).

In addition to the “classical” medial-tract PNs, two of the new types presented here, Pm_e and Pm_f, displayed completely different morphologies reflected in multiglomerular innervations in the AL and an atypical projection pattern in the protocerebrum. The glomerular ramifications of the Pm_e type, characterized by smooth neural processes enveloping each of several glomeruli, have - as far as we know - not been reported previously. Such a branching pattern may suggest a direct synaptic input from the sensory terminals that in moths are shown to target only the peripheral part of the glomeruli (Christensen et al., 1995; Zhao et al., 2016). Another interesting observation is the cell body location of the Pm_e neuron in the anterior cell cluster, which is reported to contain 16 somata in *M. sexta* (Homberg et al., 1988). Only a few PNs linked to this small cluster have been previously described, all being uniglomerular (Sun et al., 1997).

The other multiglomerular medial-tract neuron, Pm_f, innervated the AL in a completely different manner; as shown in **Figure 3**, its ramifications covered densely the entire region of the posterior AL, actually forming a non-glomerular arborization pattern. This area may include an assembly of newly discovered glomeruli in heliothine moths (Zhao et al., 2016). Interestingly, one posteriorly located glomerulus in *Drosophila* was recently found to receive input about humidity (Enjin et al., 2016). The most remarkable characteristic of the neuron identified here is that it projected to the contralateral hemisphere exclusively, omitting innervation of the Ca and the LH.

Generally, the target areas of the multiglomerular PNs described here differ from those of the “classical” uniglomerular PNs by including diverse neuropils such as the inferior neuropil, the posterior lateral protocerebrum, plus the ventro-lateral, ventro-medial, and superior neuropil. The different terminal regions of the uni- and multiglomerular medial-tract PNs indicate their participation in different neural circuits serving distinct functions. The morphology of the multiglomerular PNs suggest that they fill another purpose than processing signals related to odor identification, for example arousal or suppression. Also, we cannot exclude the possibility that some of these neurons carry information from higher brain regions into the antennal lobe and should thus be classified as centrifugal neurons.

As mentioned in the results, we did not find any medial-tract PNs similar to those classified as PIb and PIc in *M. sexta* (Homberg et al., 1988). Since the PIb type was reported to possess very thin fibers, assumingly belonging to sensory neurons, it is quite unlikely that this neuron category would have been identified via the intracellular recording technique used in the study presented here. The other neuron type, classified as PIc, was described to “leave the IACT laterally, between the central body and the calyces.” This type may therefore project in the newly identified tALT and correspond to the Pt neuron in our study.

#### Projection Neurons Passing in the lALT

Altogether, the lALT appears to possess the morphologically most diverse assembly of PNs. The six lateral-tract neurons identified here include five different categories, two of which correspond with types previously reported in moths (Homberg et al., 1988; Rø et al., 2007). This variation is compatible with previous data from mass staining experiments visualizing the lALT as a more diffuse pathway, with widespread terminal areas, in comparison with the other main tracts, which appear relatively congregated. A characteristic feature of two types of lateral-tract neurons identified here, Pl_a_uni and Pl_a_bi, is their projections to the superior intermediate protocerebrum, located between the AOTU and the alpha-lobe, here named the column (**Figure 3**). Previous studies have reported on this neuron category in both mass staining (Homberg et al., 1988) and single neuron staining experiments (Rø et al., 2007), then named POa. The former findings plus recent mass-staining results (Ian et al., 2016) clearly demonstrate that this trajectory, which omits the LH completely, constitutes a highly prominent sub-branch of the lALT in moths – a fact unfortunately overlooked sometimes. The intensive labeling of this particular path, as occasionally seen in mass-stained preparations, is probably due to the numerous short processes extending from the axon of the associated neurons. This characteristic feature is clearly visible in the Pl_a_uni neuron presented in **Figure 3**. Interestingly, the bilateral neuron targeting the column, the Pl_a_bi type, innervated three glomeruli in the ipsilateral AL, one of which was the LPOG. Since the LPOG is the distinct target for sensory neurons tuned to carbon dioxide (CO_2_) (Kent et al., 1986; Zhao et al., 2013), this suggests the PN’s involvement in processing information about CO_2_. The indication that the current sub-branch of the lALT is involved in encoding this kind of information is further strengthened by the previous finding of a small assembly of lateral-tract PNs originating from the LPOG and projecting into the particular area located between the AOTU and the alpha-lobe (data not published). The innervation of the MGC, LPOG, and the ordinary glomerulus, as demonstrated in the present study, might indicate the role of this neuron in conveying integrated information about the female-produced pheromone, CO_2_, and a particular plant odorant.

Among the remaining lateral-tract PNs, the Pl_b type corresponds to a particular category termed POb by Homberg et al. (1988). In the previous study, some of these neurons were reported to possess “very large club-like terminals.” However, since none of them was selectively stained, their full morphology has remained unknown. Based on reconstruction of one such neuron, as shown in **Figure 3G**, we obtained the full overview of this neuron type. Thus, its axon projects directly to the most anterior part of the ventro-lateral neuropil. Here, it extends a few relatively short processes, one of which ends up in a prominent club-like structure. This kind of focused terminal ending, being unusual among the total assembly of AL PNs, indicates a special role of these neurons in signal processing - possibly an arrangement for fast synaptic transmission. Recently we identified a population of sound-sensitive neurons projecting from the ventral cord to the anterior part of the ventro-lateral protocerebrum in *H. virescens* (Pfuhl et al., 2014). Furthermore, in *Drosophila*, the ventral part of the lateral protocerebrum is reported to receive input from the optic lobes and to include distinct optical glomeruli (Douglass and Strausfeld, 1998; Strausfeld et al., 2007; Ito et al., 2013). If the Pl_b neurons overlap with a corresponding set of optic glomeruli in the moth brain, or possibly with auditory neurons, these lateral-tract PNs may be involved in integrating odor input with information from other sensory modalities. Also, a descending odor-responding neuron with dense arborizations in the ventro-anterior area of the lateral protocerebrum, previously found in the *H. virescens* female, should be mentioned here (Løfaldli et al., 2012). Whether there is a direct connection between AL PNs and brain output neurons in this area, possibly constituting a fast path to the ventral cord, is an open question.

Generally, the lALT of moths seems to consist of mainly morphologically distinct types of PNs compared to those reported in other insects. A comparison of the lateral-tract PNs in the moth and the fruit fly, for example, demonstrates substantial differences. Actually, none of the lateral-tract neurons identified here was found in *Drosophila* (Tanaka et al., 2012). The only type of lALT PN described by Tanaka et al. (2012) that is morphologically identical to those reported in moths, is a category innervating the LH and the Ca (Homberg et al., 1988; Rø et al., 2007). Such a morphological discrepancy contrasts with the inter-specific homology applying to the mALT, and it may indicate functionally different roles of the lALT PNs across the species stressing their behavioral distinctiveness.

#### Projection Neurons Passing in the mlALT

Morphologically, the individual mlALT PNs seem to be more homogeneous than the lALT PNs. The previously described neurons confined to the mlALT were not categorized into distinct groups (Homberg et al., 1988; Rø et al., 2007). However, based on the axonal outputs of the mlALT PNs, our results indicate the presence of at least two morphological types: the first type, Pml_a, targets the LH exclusively whereas the second type, Pml_b, sends collaterals to additional areas like the superior lateral protocerebrum, the posterior lateral protocerebrum, and the base of the Ca. Previous studies in different insect species have reported a substantial proportion of GABAergic neurons in this tract (moth: Hoskins et al., 1986, honeybee: Schäfer and Bicker, 1986, fruit fly: Okada et al., 2009). In the heliothine moth, the number is estimated to be in the range of 40–70 fibers (Berg et al., 2009). In total, the mlALT is reported to contain ∼120 axons (Homberg et al., 1988). Whether the GABAergic PNs include both or only one of the neuron types described here is not yet known.

### Overlapping vs. Non-overlapping Output Areas

The registration of single neurons obtained from different individuals into the standard atlas of the male *H. virescens* brain allowed for a more detailed comparison of the target regions of PNs confined to different tracts. We have previously performed a comparison of physiologically distinct types of PNs confined to one tract in this species (Zhao et al., 2014). Based on the PNs that could be reconstructed and registered into the atlas in the study presented here, we were able to compare the projection patterns of PNs confined to two different tracts, the mALT and mlALT. Interestingly, as shown in **Figure 9**, the axon terminals of the main types of PNs passing in the medial and the mediolateral ALT, respectively, show almost complete overlap in the LH. The congruence of the neuron terminals originating from the two tracts is visible in all orientations of the model.

This somewhat contradicts previous findings in *H. virescens*. Thus, Rø et al. (2007) claimed that the spatial separation of the intracellularly stained PNs in the different tracts is largely maintained in the secondary olfactory centers. However, this study did not include a detailed anatomical comparison by means of a standardized reference brain to which individually stained neurons were fitted. Studies of *M. sexta*, on the other hand, have reported that mlALT PNs originating from the MGC and the ordinary glomeruli, respectively, overlap with terminals of medial-tract PNs in the two relevant LH regions (Homberg et al., 1988). Interestingly, previous findings from *Drosophila* have demonstrated the cooperation between PNs confined to the medial and mediolateral ALT, respectively, both as concerns connectivity and physiology (Liang et al., 2013; Wang et al., 2014). Here, third order LH neurons are assumed to receive input from both types of antennal-lobe PNs, confined to the medial and the mediolateral ALT, respectively. Furthermore, convergent inhibitory and excitatory input in the LH of the fruit fly is reported to play a role in processing features such as hedonic valence and intensity (Strutz et al., 2014). Based on the present and previous data, an arrangement including input from two cooperative olfactory tracts - one inhibitory and one excitatory - seems to apply to moths as well.

Generally, the PNs confined to the lALT seem to target regions other than that innervated by the medial and the medio-lateral ALT. Of the six lateral-tract neurons identified here, only one terminated in the LH. Fortunately, this particular PN was stained simultaneously with a uniglomerular medial-tract neuron and we could therefore compare the two neurons’ target regions in the LH directly from the confocal images. As shown in **Figure 10**, including a reconstruction of the confocal stack, there are no overlapping terminal areas of the two PN types. Since the remaining six lateral-tract neurons projected to protocerebral regions outside the LH, we can conclude that none of the lALT PNs identified here form overlapping terminal regions with those of the main neuron types confined to the mALT/mlALT.

In order to fully understand the purpose of an arrangement including chemosensory signal transfer along parallel antennal-lobe tracts, such as described in the moth brain, more data on morphological and physiological properties characterizing PNs confined to the distinct paths have to be obtained. Based on the results presented here, however, we suggest that the mALT and mlALT, being part of one neural circuit, are involved in cooperative information transfer to third order neurons. The lALT, on the other hand, terminating in several protocerebral areas distinct from the output areas of the m- and mlALT, seems to participate in other neural circuits (**Figure 11**).

## Author Contributions

EI: Conception and design of the work, data acquisition, analysis, interpretation of data; drafting the work, revising and approval of the version to be published. XZ: Conception and design of the work, data acquisition, analysis, revising and approval of the version to be published. AL: Data acquisition, analysis, drafting the work, revising and approval of the version to be published. BB: Conception and design of the work, analysis, interpretation of data; drafting the work, revising and approval of the version to be published.

## Conflict of Interest Statement

The authors declare that the research was conducted in the absence of any commercial or financial relationships that could be construed as a potential conflict of interest.
