# Supplementary material for: Individual Neurons Confined to Distinct Antennal-Lobe Tracts in the Heliothine Moth: Morphological Characteristics and Global Projection Patterns
Source: Front Neuroanat. 2016 Oct 24;10:101. doi: 10.3389/fnana.2016.00101 (PMC5075568; doi:10.3389/fnana.2016.00101)
Supplement: Supplementary file 1 [file Table_1.DOCX]

**Explanation of demarcation**

In general, the boundaries are specified in accordance with the system used by Ito et al. (2014).

Main neuropil regions^[[1]](#footnote-1)^ include:

- **optic lobe (OL)**
- **mushroom body (MB)** including the calyx (CA), the pedunculus (PED), the alpha lobe (αL), and the beta lobe (βL)
- **central complex (CX)** including the central body (CB)
- **lateral horn (LH)**
- **ventrolateral neuropils (VLNP)** including the anterior optic tubercle (AOTU), the posterior lateral protocerebrum (PLP), and the ventrolateral protocerebrum (VLP) – which, in turn, includes the anterior ventrolateral protocerebrum (AVLP) and the posterior ventrolateral protocerebrum (PVLP).
- **superior neuropils (SNP)** including the superior lateral protocerebrum (SLP)
- **inferior neuropils (INP)**
- **ventromedial neuropils (VMNP)**
- **antennal lobe (AL)**
- **gnathal ganglion (GNG)**

The following neuropil regions, which are clearly visualized by synapsine immunostaing and/or their obvious peripheral demarcation, are specified in the standard brain atlas: OL, CA, PED, αL, CB, AOTU, AL, and GNG.

The remaining neuropil regions of relevance here, which were not obviously delineated in the synapsine-labeled preparations, called *unspecified neuropils*, were stated as follows:

- The LH was defined as the neuropil region in the lateral protocerebrum receiving terminal branches from the main category of AL PNs confined to the medial and mediolateral ALT, respectively, plus a minor category of PNs passing in the lateral ALT. The inferior border of the LH adjoins the VLNP. Medially, the LH borders the INP, and superiorly, the SLP of the SNP. Its remaining superior and lateral border makes up the brain surface in this region.
- The VLNP, which consists of the VLP and the PLP, is located most laterally in the ventrolateral cerebrum. The VLP, which is positioned between the AL, the LAL, and the OL, is located anteriorly of the great commissure whereas the PLP is located posteriorly of this prominent path. The VLP, in turn, is divided, by the lateral AL‑tract, into the AVLP and PVLP. The inferior border of the VLNP adjoins the Wedge (WED; a region not indicated here) and its superior border the AOTU, the SLP of the SNP, and the LH. The anterior border of the AVLP forms the surface of the central brain in this region whereas the PLP forms a posterior brain surface. Medially, the VLNP has a boundary against the LAL, the PED, the VMNP, and the INP.
- The SNP includes the most superior neuropils of the central brain. The most lateral area of the SNP, in turn, named the SLP, borders the lateral part of the Ca, the medial part of the LH, and the posterior part of the AOTU (the medial border of the SLP could not be accurately defined). Laterally and posteriorly, the SLP forms the surface of the current brain region. Regarding the remaining SNP area, it extends to the brain midline medially and forms the surface of the central brain anteriorly and posteriorly. Inferiorly, the SNP borders with the VLP, the PLP, the INP, and the CB.
- The INP is positioned below the SNP, nearby the PED, the βL, and the upper part of the CX. Anteriorly, a part of the INP makes up the surface of the current brain region. Laterally, it borders the SLP and medially, the LAL (not shown), plus the AVLP. Posteriorly, the INP borders the SNP, the mALT, and the cell body rind. Superiorly, the INP borders the SNP and inferiorly, the AL and the LAL. Medially, the INP borders the CB and extends to the brain midline. Laterally, the INP borders the SLP, the LH, and the VLNP.
- The VMNP is located below the INP, medially to the VLNP, and laterally to the esophageus. Anteriorly, this region borders the AL, the LAL, and the wedge. Posteriorly, parts of the VMNP make up the surface of the current brain region. Superiorly, the VMNP borders the mALT, plus the INP, and inferiorly, the great commissure and the GNG.

1. Neuropil supercategories in **red bold**. [↑](#footnote-ref-1)
